# Risks and use of ERCP during the diagnostic workup in a national cohort of biliary cancer

**DOI:** 10.1007/s00464-024-11449-8

**Published:** 2024-12-13

**Authors:** Anna Forslund, Erik Haraldsson, Erik Holmberg, Peter Naredi, Magnus Rizell

**Affiliations:** 1 Transplant Institute, Sahlgrenska University Hospital, 41345 Gothenburg, Sweden; 2 Department of Surgery, Sahlgrenska University Hospital, Gothenburg, Sweden; 3 Department of Oncology, Institute of Clinical Sciences, University of Gothenburg, Gothenburg, Sweden; 4 Department of Surgery, Institute of Clinical Sciences, University of Gothenburg, Gothenburg, Sweden

**Keywords:** ERCP, Biliary cancer, Bile duct cancer, Complications, PEP, Cholangitis

## Abstract

**Background:**

In biliary cancer, the indication of endoscopic intervention might be diagnostic as well as therapeutic, in the latter situation with the aim to relieve biliary obstruction e.g. by stenting. Our aim was to investigate the use of endoscopic biliary interventions during the diagnostic workup of biliary cancers in a national cohort, and to evaluate their complications, especially cholangitis and pancreatitis.

**Methods:**

This is a registry-based study of national cohort of patients with biliary cancers in Sweden 2010–2020. The use of endoscopic retrograde cholangiopancreatography (ERCP) during the diagnostic work up period before treatment onset, and risk of complications were evaluated in patients with gallbladder cancer, intrahepatic-, perihilar- and distal cholangiocarcinoma. The risk of complications was compared depending on age, sex, comorbidity, in relation to stage and curative intent, endoscopy unit size, and with relation to survival.

**Results:**

Forty percent of the patients with biliary cancer underwent ERCP during the diagnostic workup, with variations depending on diagnosis. There was a 20% overall risk of periprocedural complications, a 9% risk of post-ERCP-pancreatitis (PEP), and a 6% risk of cholangitis. Increasing tumor stage did not increase risk, nor did comorbidity. The complication rates were slightly higher for younger patients and those undergoing curative treatment. For perihilar cholangiocarcinoma (pCCA) treated with curative intention, the risk of periprocedural complications was as high as 30.7%. No association between post-ERCP complications and survival was found.

**Conclusion:**

Irrespective of type of biliary cancer, ERCP is frequently used during diagnostic workup. The complication risk indicates that primary biliary cancers are complication prone, regardless of stage. Notably the risk of complications was the highest for younger patients with low comorbidity scores, as well as for patients undergoing curatively aiming treatment.

**Supplementary Information:**

The online version contains supplementary material available at 10.1007/s00464-024-11449-8.

Endoscopic retrograde cholangiopancreatography (ERCP) is commonly used for both diagnostic and therapeutic procedures in the biliary tree. It is easily accessible, and in Sweden the number of ERCP is around 9000/year in 10 million inhabitants [1]. However, ERCP is an invasive intervention, and complications continue to be reported. Risk factors for post-ERCP-pancreatitis (PEP) include female sex and difficult biliary cannulation [2, 3], while cholangitis is commonly associated with factors such as hilar obstruction [2] and malignancy [4].

Obstructive jaundice is a frequent indication for ERCP in biliary cancers, not only in perihilar cholangiocarcinoma (pCCA) and distal (dCCA) cholangiocarcinoma [5, 6], but also in gallbladder cancer (GBC) and intrahepatic cholangiocarcinoma (iCCA). Jaundice in e.g., GBC may signal a more advanced stage, but if an early or advanced stage in itself impacts the risk for complications post-ERCP is less explored [5–9].

Our aim was to investigate the use of ERCP for biliary cancers, as well as associated complications, during the diagnostic workup before treatment onset. We were questioning if type of biliary cancer, patient-related factors and size of endoscopy unit may impact risk of complications, specifically pancreatitis.

## Materials and methods

A national cohort, for the years 2010–2020, was identified by extracting data from the National Registry for Liver, Bile Duct and Gallbladder Cancer (SweLiv [10]) and National Registry for Pancreatic and Periampullary Cancer (Pancreatic Register [11]). The coverage of the registries is reported to be 92–96% in relation to the national cancer register [12, 13].

The registries use the ICD-10 codes C22.1 for iCCA, C23.9 for GBC, C24.0 for pCCA, and C24.0 for dCCA. Biliary cancer with overgrowth, multiple foci in the in biliary tract (C24.8 and C24.9) as well as those with discrepancies in regard to code number between the registries were grouped together as “otherCCA”.

Locally advanced disease was defined as T-stage 4 for iCCA. For GBC, dCCA and pCCA as T-stage 3 or worse although we for pCCA also included Bismuth IV. The concept group “otherCCA” were all considered as locally advanced. Patients who underwent resection, irrespective of whether radicality was achieved in the pathology report, was defined as “curatively” treated.

Patients where the initial diagnosis suggested malignancy but final diagnosis was benign were excluded. Patients with malignancy found incidentally at surgery were excluded.

The national biliary cancer cohort was matched with patients with biliary endoscopic interventions and their complications, as registered in GallRiks [14] the Swedish Registry for Gallstone Surgery and ERCP. The coverage of GallRiks is reported to be 90%, when validated against the Swedish National Patient Registry [15, 16].

**Fig. 1:**
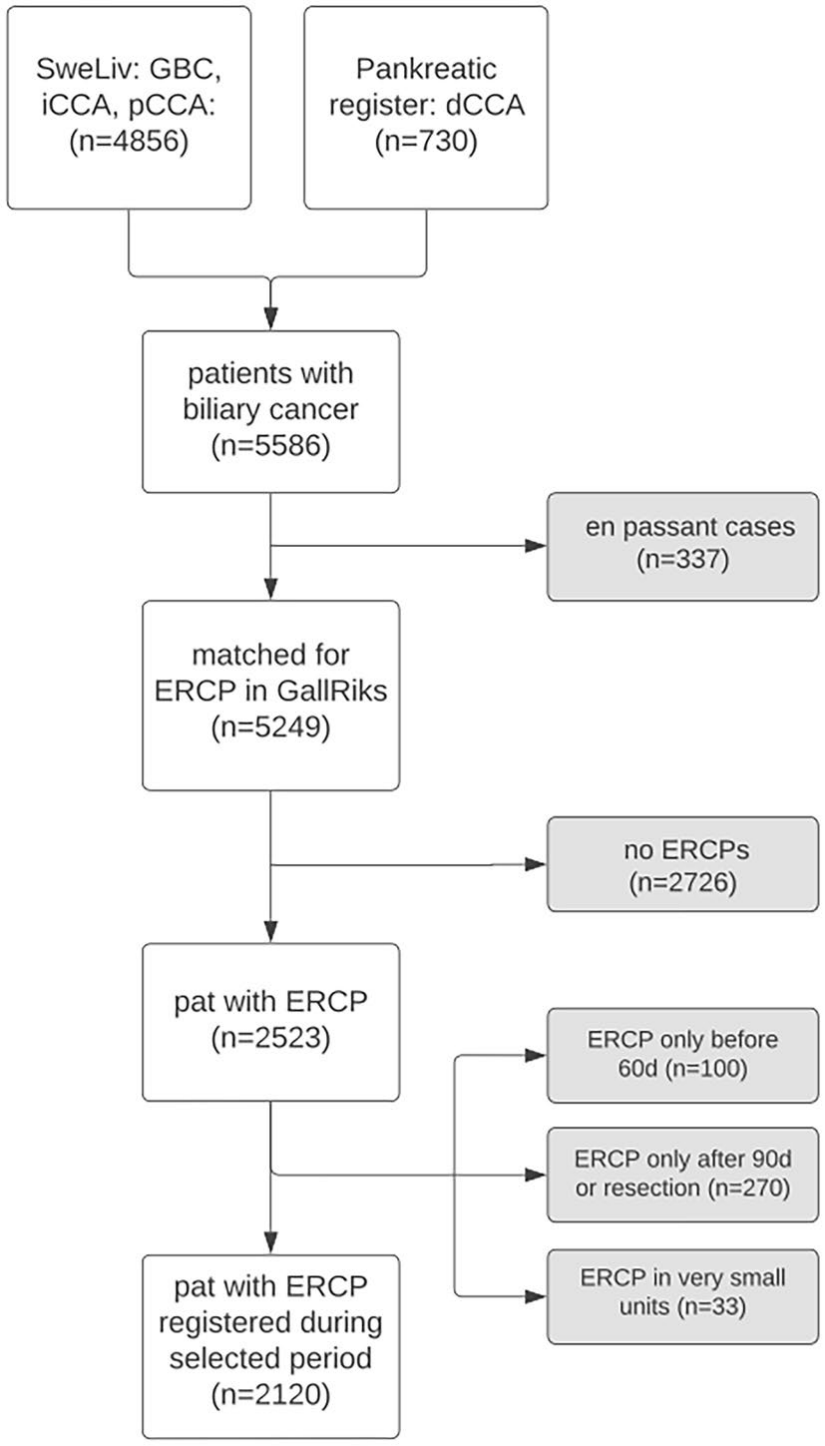
Biliary cancer cohort diagnosed between 2010 and 2020. Excluded patients in grey boxes

Date of diagnosis was defined as the earliest date of either radiology or pathology, as defined in the registries. We included biliary endoscopies undertaken 60 days before date of diagnosis until date of curatively aiming treatment, or for palliative patients, up to 90 days after diagnosis (Fig. 1).

However, for descriptive purposes, endoscopic interventions prior to, and after, the selected time period were noted.

Complications (within 30 days) were investigated in relation to the first endoscopy. All ERCPs, even if only an attempt with unsuccessful cannulation were included.

Complications include intra- or postprocedural complications like bleeding (requiring blood transfusion or reintervention) and perforation (contrast leakage or intestinal perforation), pancreatitis and cholangitis. Cholangitis (biliary infection requiring hospitalization/biliary sepsis) and post-ERCP-pancreatitis (PEP) (abdominal pain > 24h and S-amylase > × 3), are registered only if they require hospitalization. All complications that were reported only as a written comment, were scrutinized and in applicable cases re-sorted into already existing categories. Complications such as prolonged hospital stay without any clear cause or urinary tract infections are not included in this report.

Neither persistent jaundice nor stent dysfunction were studied, and hence not defined as a complication in this study. We did not register the need of a new endoscopy as a complication, nor did we have access to any bilirubin levels. Sedation-related complications were not considered.

### Statistics

Where appropriate, Fisher’s exact test, Chi-squared test, ANOVA, and Kruskal–Wallis equality-of-populations rank test were performed for comparisons of variable distributions between diagnostic groups. The effects of patient-, diagnostic-, and treatment factors on different complication types were analyzed using univariable and multivariable Poisson regression, and incidence rate ratios (IRR) with 95% confidence intervals (CI) were estimated. Variables significant at *p* < 0.2 in univariable analyses, along with previously statistically significant prognostic factors, were tested in multivariable analyses through manual elimination to assess their confounding effect on complications.

The Kaplan–Meier method was employed to calculate overall survival with 95% CI, with time counted from the date of diagnosis to the date of death or censored at the last follow-up date. The log-rank test was used for comparison between survival curves. A two-sided *p*-value of < 0.05 was considered statistically significant. Statistical analysis was performed with Stata version 18.0 for Mac (StataCorp. 2023, College Station, TX: StataCorp LLC).

## Results

Data were collected in September 2022 from the Pancreatic register and in December 2022 from SweLiv. At the time for data extraction, vital status was collected from the National Population Register. The data extraction from GallRiks was made in June 2023.

Out of a total of 5249 patients (Fig. 1), the percentage of biliary cancers that underwent ERCP was 85% of the dCCA, 73% of the pCCA, 32% of the GBC, 29% iCCA and 56% of those that were diagnosed as otherCCA.

After selection according to the inclusion/exclusion criteria above (Fig. 1), 2120 patients undergoing ERCP in the selected period were included. 68% of them underwent one ERCP, 25% underwent two ERCPs, and 7% underwent three or more (3013 ERCPs in total). Patients were included even if they were not ERCP naïve, and 179 patients (8.4%) had another ERCP registered at any time before the selected time period.

The number of ERCPs performed per center (hospital) in this cohort, varied between one and 231 among the 51 hospitals. Hospitals performing on average less than one ERCP on malignant indication per year were excluded from the cohort (9 out of 51 hospitals and in total 33 patients). The hospitals were divided into two groups based on the total number of ERCPs performed on patients with biliary cancer to investigate differences between high- and low volume centers (1063 vs 1057 patients with 10 vs 32 centers),

There were 235 (11%) failed ERCPs in our cohort, mostly due to unsuccessful cannulations (*n* = 197 (9% of 2120)). 108 (46%) of those underwent another ERCP later on. The periprocedural complication rate for the 197 patients with unsuccessful cannulation was 22.3%.

For the 107 special procedures reported as first intervention, including 90 cholangioscopies and one intraductal ultrasound, a 18.7% complication rate was found.

Descriptive data including patient characteristics, diagnosis, stage, treatment and stenting are shown in Table 1.

**Table 1:** Patients undergoing ERCP by diagnosis

	GBC	ICCA	pCCA	dCCA	Other	Total	*p*-value
	*N* = 481	*N* = 367	*N* = 537	*N* = 563	*N* = 172	*N* = 2120	
Age, median (IQR)	72.0 (66.0–79.0)	70.0 (62.0–76.0)	72.0 (64.0–78.0)	72.0 (65.0–78.0)	72.5 (63.5–80.0)	71.0 (65.0–78.0)	0.002
Age group							0.019
< 60	66 (13.7%)	74 (20.2%)	83 (15.5%)	70 (12.4%)	25 (14.5%)	318 (15.0%)	
60–75	255 (53.0%)	197 (53.7%)	269 (50.1%)	307 (54.5%)	82 (47.7%)	1110 (52.4%)	
> 75	160 (33.3%)	96 (26.2%)	185 (34.5%)	186 (33.0%)	65 (37.8%)	692 (32.6%)	
Sex							< 0.001
Male	165 (34.3%)	192 (52.3%)	292 (54.4%)	302 (53.6%)	72 (41.9%)	1023 (48.3%)	
Female	316 (65.7%)	175 (47.7%)	245 (45.6%)	261 (46.4%)	100 (58.1%)	1097 (51.7%)	
ASA grp							< 0.001
1	44 (9.1%)	41 (11.2%)	65 (12.1%)	83 (14.7%)	19 (11.0%)	252 (11.9%)	
2	247 (51.4%)	181 (49.3%)	292 (54.4%)	328 (58.3%)	88 (51.2%)	1136 (53.6%)	
3–4	190 (39.5%)	145 (39.5%)	180 (33.5%)	152 (27.0%)	65 (37.8%)	732 (34.5%)	
Locally advanced							< 0.001
No	68 (14.1%)	240 (65.4%)	217 (40.4%)	117 (20.8%)	0 (0.0%)	642 (30.3%)	
Yes	371 (77.1%)	61 (16.6%)	243 (45.3%)	124 (22.0%)	172 (100.0%)	971 (45.8%)	
Missing	42 (8.7%)	66 (18.0%)	77 (14.3%)	322 (57.2%)	0 (0.0%)	507 (23.9%)	
Curative treatment							< 0.001
No	422 (87.7%)	297 (80.9%)	403 (75.0%)	164 (29.1%)	150 (87.2%)	1436 (67.7%)	
Yes	59 (12.3%)	70 (19.1%)	134 (25.0%)	399 (70.9%)	22 (12.8%)	684 (32.3%)	
Hospital							0.005
High volume	251 (52.2%)	163 (44.4%)	245 (45.6%)	306 (54.4%)	92 (53.5%)	1057 (49.9%)	
Low volume	230 (47.8%)	204 (55.6%)	292 (54.4%)	257 (45.6%)	80 (46.5%)	1063 (50.1%)	
Stenosis							< 0.001
No	122 (25.4%)	72 (19.6%)	76 (14.2%)	128 (22.7%)	23 (13.4%)	421 (19.9%)	
Below cystic duct	70 (14.6%)	29 (7.9%)	59 (11.0%)	374 (66.4%)	23 (13.4%)	555 (26.2%)	
Above cystic duct	289 (60.1%)	266 (72.5%)	402 (74.9%)	61 (10.8%)	126 (73.3%)	1144 (54.0%)	
Stenting							0.36
No	176 (36.6%)	145 (39.5%)	191 (35.6%)	231 (41.0%)	64 (37.2%)	807 (38.1%)	
Yes	305 (63.4%)	222 (60.5%)	346 (64.4%)	332 (59.0%)	108 (62.8%)	1313 (61.9%)	

Data based on first ERCP

### Periprocedural complications

Out of the 2120 patients, 49 (2.3%) experienced intraprocedural complications (bleeding, contrast- or bile leakage). 13 of them (26.5%) also had additional post-procedural complications.

**Fig. 2:**
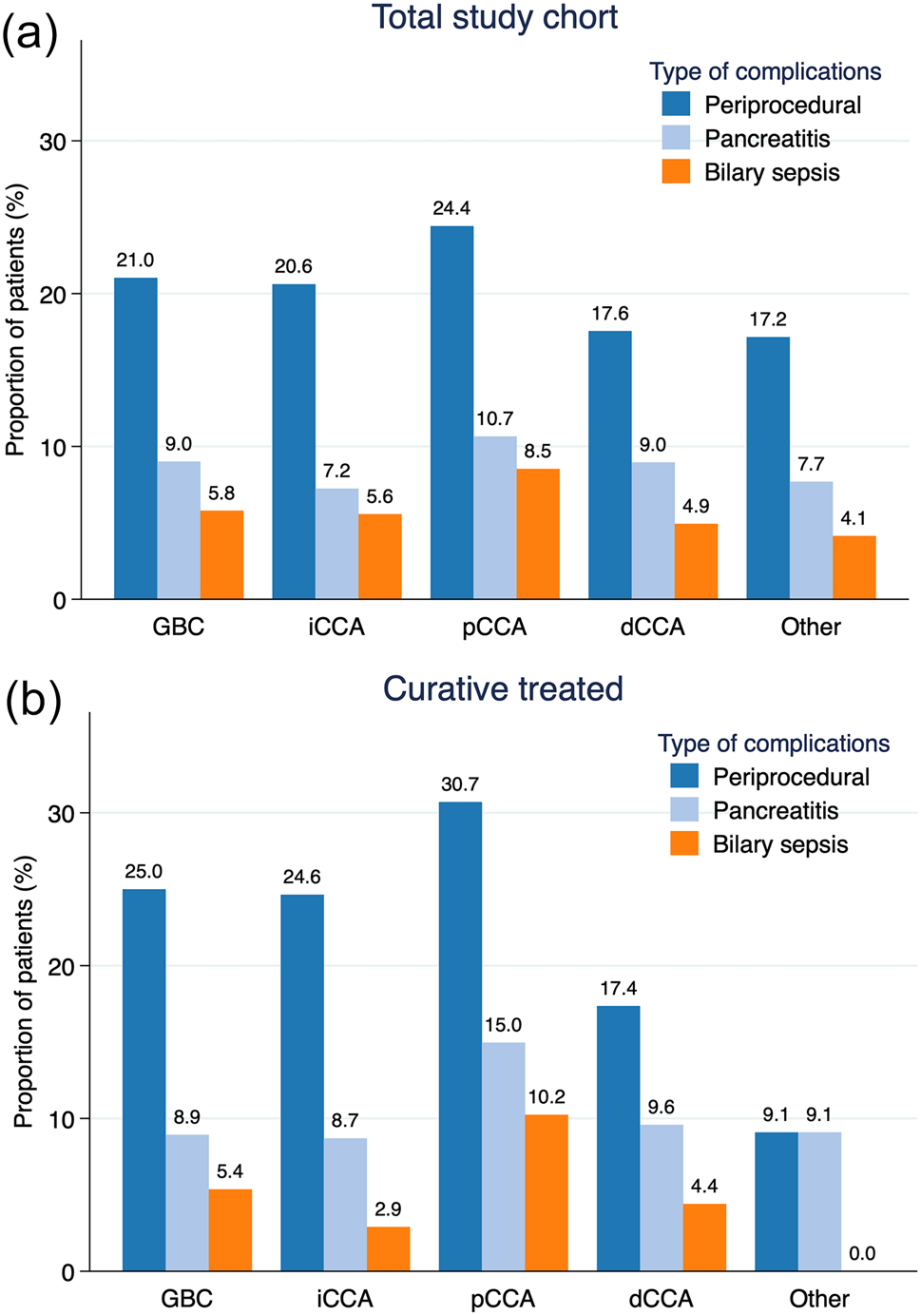
**a** Peri-procedural complications total. **b** Peri-procedural complications curative intention

387 patients (18.8%, *p* = 0.025) had post-procedural complication after first ERCP (within 30 days), see Table 2, and Fig. 2a.

**Table 2:** Intraprocedural and 30-day postprocedural complications grouped by diagnosis

	GBC	iCCA	pCCA	dCCA	Other	Total	*p*-value*
	*N* = 466	*N* = 359	*N* = 516	*N* = 547	*N* = 169	*N* = 2057	
Periprocedural complications							0.064
No	368 (79.0%)	285 (79.4%)	390 (75.6%)	451 (82.4%)	140 (82.8%)	1634 (79.4%)	
Yes	98 (21.0%)	74 (20.6%)	126 (24.4%)	96 (17.6%)	29 (17.2%)	423 (20.6%)	
Intraprocedural total							0.051
No	455 (97.6%)	343 (95.5%)	509 (98.6%)	537 (98.2%)	164 (97.0%)	2008 (97.6%)	
Yes	11 (2.4%)	16 (4.5%)	7 (1.4%)	10 (1.8%)	5 (3.0%)	49 (2.4%)	
Intraprocedural bleeding							0.34
No	464 (99.6%)	356 (99.2%)	514 (99.6%)	544 (99.5%)	166 (98.2%)	2044 (99.4%)	
Yes	2 (0.4%)	3 (0.8%)	2 (0.4%)	3 (0.5%)	3 (1.8%)	13 (0.6%)	
Intraprocedural leakage/bile leakage							0.075
No	457 (98.1%)	346 (96.4%)	511 (99.0%)	539 (98.5%)	167 (98.8%)	2020 (98.2%)	
Yes	9 (1.9%)	13 (3.6%)	5 (1.0%)	8 (1.5%)	2 (1.2%)	37 (1.8%)	
Postprocedural complications total							0.025
No	376 (80.7%)	295 (82.2%)	396 (76.7%)	459 (83.9%)	144 (85.2%)	1670 (81.2%)	
Yes	90 (19.3%)	64 (17.8%)	120 (23.3%)	88 (16.1%)	25 (14.8%)	387 (18.8%)	
Postprocedural bleeding							0.090
No	452 (97.0%)	353 (98.3%)	508 (98.4%)	543 (99.3%)	167 (98.8%)	2023 (98.3%)	
Yes	14 (3.0%)	6 (1.7%)	8 (1.6%)	4 (0.7%)	2 (1.2%)	34 (1.7%)	
Postprocedural leakage							0.81
No	456 (97.9%)	349 (97.2%)	504 (97.7%)	537 (98.2%)	167 (98.8%)	2013 (97.9%)	
Yes	10 (2.1%)	10 (2.8%)	12 (2.3%)	10 (1.8%)	2 (1.2%)	44 (2.1%)	
Postprocedural pancreatitis (PEP)							0.51
No	424 (91.0%)	333 (92.8%)	461 (89.3%)	498 (91.0%)	156 (92.3%)	1872 (91.0%)	
Yes	42 (9.0%)	26 (7.2%)	55 (10.7%)	49 (9.0%)	13 (7.7%)	185 (9.0%)	
Postprocedural cholangitis							0.12
No	439 (94.2%)	339 (94.4%)	472 (91.5%)	520 (95.1%)	162 (95.9%)	1932 (93.9%)	
Yes	27 (5.8%)	20 (5.6%)	44 (8.5%)	27 (4.9%)	7 (4.1%)	125 (6.1%)	
Postprocedural other							0.58
No	448 (96.1%)	351 (97.8%)	500 (96.9%)	533 (97.4%)	166 (98.2%)	1998 (97.1%)	
Yes	18 (3.9%)	8 (2.2%)	16 (3.1%)	14 (2.6%)	3 (1.8%)	59 (2.9%)	
30d mortality							< 0.001
No	407 (87.3%)	319 (88.9%)	484 (93.8%)	528 (96.5%)	146 (86.4%)	1884 (91.6%)	
Yes	59 (12.7%)	40 (11.1%)	32 (6.2%)	19 (3.5%)	23 (13.6%)	173 (8.4%)	
90d mortality							< 0.001
No	312 (67.0%)	248 (69.1%)	418 (81.0%)	486 (88.8%)	101 (59.8%)	1565 (76.1%)	
Yes	154 (33.0%)	111 (30.9%)	98 (19.0%)	61 (11.2%)	68 (40.2%)	492 (23.9%)	

*Fisher’s exact test

63 patients with missing values on postprocedural complication are excluded

A total of 20.6% experienced any periprocedural complication (intra- or postprocedural). When analyzed per diagnosis, pCCA had a periprocedural complication rate of 24.4% to be compared with 19.3% for all other biliary cancers (*p* = 0.064, Table 2). For postprocedural complications, the differences between diagnoses were significant (range 14.8–23.3% *p* = 0.025).

For curatively treated patients the periprocedural complication rate was 21.1% (range 9.1–30.7% *p* = 0.011, between diagnoses). There were also differences in postprocedural complication rates between the diagnoses. For both peri- and postprocedural complications, the rate was the highest for pCCA (Table 2 in supplements). For palliative patients, the periprocedural complication rate was 20.3% (range 18.0–22.4%) (Table 1a–b in supplements).

23.2% of the patients < 60 years old in the total cohort suffered complications compared to 20.3% of the patients between 60–75 and 19.8% of the patients > 75 years but neither of these differences were statistically significant (Table 3).

**Table 3:** Peri-procedural complications

	ERCP cohort *N* = 2057 *n* (%)	Complications *N* = 423 *n* (%)	Univariable Poisson regression IRR (CI 95%)	*P*	Multivariable Poisson regression IRR (CI 95%)	*P*
*Age group*						
< 60	310 (15.1%)	72 (23.2%)	Ref			
60–75	1074 (52.2%)	218 (20.3%)	0.87 (0.67–1.14)	0.322		
> 75	673 (32.7%)	133 (19.8%)	0.85 (0.64–1.13)	0.270		
*Sex*						
Male	989 (48.1%)	208 (21.0%)	Ref			
Female	1068 (51.9%)	215 (20.1%)	0.96 (0.79–1.16)	0.653		
*ASA grp*						
1	248 (12.1%)	63 (25.4%)	Ref			
2	1102 (53.6%)	237 (21.5%)	0.85 (0.64–1.12)	0.240		
3–4	707 (34.4%)	123 (17.4%)	0.68 (0.51–0.93)	0.015		
*Diagnosis*						
GBC	466 (22.7%)	98 (21.0%)	Ref			
ICCA	359 (17.5%)	74 (20.6%)	0.98 (0.72–1.33)	0.896		
pCCA	516 (25.1%)	126 (24.4%)	1.16 (0.89–1.61)	0.267		
dCCA	547 (26.6%)	96 (17.6%)	0.83 (0.63–1.11)	0.208		
Other	169 (8.2%)	29 (17.2%)	0.82 (0.54–1.23)	0.336		
Locally advanced*						
No	623 (30.3%)	138 (22.2%)	Ref			
Yes	946 (46.0%)	186 (19.7%)	0.89 (0.71–1.11)	0.289		
Missing	488 (23.7%)	99 (20.3%)				
*Curative treatment*						
No	1397 (67.9%)	284 (20.3%)	Ref			
Yes	660 (32.1%)	139 (21.1%)	1.04 (0.85–1.27)	0.733		
Hospital size*						
High volume	1026 (49.9%)	192 (18.7%)	Ref			
Low volume	1031 (50.1%)	231 (22.4%)	1.20 (0.99–1.45)	0.065		
Stenting*						
No	782 (38.0%)	166 (21.2%)	Ref			
Yes	1275 (62.0%)	257 (20.2%)	0.95 (0.78–1.15)	0.658		
Stenosis*						
No	407 (19.8%)	88 (21.6%)	Ref			
Below cystic duct	540 (26.2%)	97 (18.0%)	0.83 (0.62–1.11)	0.208		
Above cystic duct	1110 (54.0%)	238 (21.4%)	0.99 (0.78–1.27)	0.947		

*Not included in multivariate analysis

ASA score (American Society of Anesthesiologists) 3–4 was reported to have significantly less periprocedural complications (*p* = 0.015) than ASA score 1 in univariate analysis (Table 3). For palliative patients, ASA class 1 had a complication rate of 32.8% whereas ASA 3–4 and ASA 2 had significantly less complications than at 17.4% (*p* = 0.001) and 20.5% (*p* = 0.007), respectively, (Table 3a in supplements).

There was no difference in periprocedural complications whether stenting was performed or not. More complications were registered in high volume ERCP centers at 22.4% compared to 18.7% for low volume centers, though not statistically significant (*p* = 0.065). Larger centers had more PEP at 10.5% compared to 7.5% (*p* = 0.025) in univariate analysis.

### Cholangitis/sepsis

In total 5.9% of the patients suffered post-procedural cholangitis. For curative patients the rate was 5.1% and for palliative 6.3%.

No difference was seen between age groups in the total cohort. However, patients with ASA score 3–4 had significantly less cholangitis at 4.4% compared to 9.3% for ASA 1, in univariate analysis (*p* = 0.006).

For palliative patients, ASA score 3–4 had less cholangitis than ASA score 1 (4.3% compared to 12.5% *p* = 0.001 in univariate analysis). For curative patients there was no significant difference in ASA score. For both the palliative and curative group there was no significant difference between age groups, sex or diagnosis. Patients with pCCA had a numerically higher rate of cholangitis than other diagnoses though not statistically significant.

### Pancreatitis

For the total cohort the PEP rate was 9.0% (10.5% curatively treated, 8.3% palliative). 13.9% of patients < 60 years old in the total cohort suffered from PEP. The PEP rates for age group > 75 years was 7.3% and for 60–75 years 8.7% (differences significant in multivariate analysis, see Table 4).

**Table 4:** Post-procedural pancreatitis

	ERCP cohort *N* = 2057 *n* (%)	PEP *N* = 185 *n* (%)	Univariable Poisson regression IRR (CI 95%)	*P*	Multivariable Poisson regression IRR (CI 95%)	*P*
*Age group*						
< 60	310 (15.1%)	43 (13.9%)	Ref		Ref	
60–75	1074 (52.2%)	93 (8.7%)	0.62 (0.43–0.90)	0.011	0.65 (0.45–0.94)	0.023
> 75	673 (32.7%)	49 (7.3%)	0.52 (0.35–0.79)	0.002	0.58 (0.38–0.88)	0.011
*Sex*						
Male	989 (48.1%)	94 (9.5%)	Ref			
Female	1068 (51.9%)	91 (8.5%)	0.90 (0.67–1.20)	0.457		
*ASA grp*						
1	248 (12.1%)	31 (12.5%)	Ref		Ref	
2	1102 (53.6%)	103 (9.3%)	0.75 (0.60–1.12)	0.156	0.83 (0.55–1.25)	0.367
3–4	707 (34.4%)	51 (7.2%)	0.68 (0.37–0.90)	0.016	0.67 (0.42–1.06)	0.088
*Diagnosis*						
GBC	466 (22.7%)	42 (9.0%)	Ref			
iCCA	359 (17.5%)	26 (7.2%)	0.80 (0.49–1.31)	0.381		
pCCA	516 (25.1%)	55 (10.7%)	1.18 (0.79–1.77)	0.413		
dCCA	547 (26.6%)	49 (9.0%)	0.99 (0.66–1.50)	0.977		
Other	169 (8.2%)	13 (7.7%)	0.52 (0.46–1.59)	0.618		
Locally advanced*						
No	623 (30.3%)	57 (9.1%)	Ref			
Yes	946 (46.0%)	87 (9.2%)	1.01 (0.72–1.40)	0.976		
Missing	488 (23.7%)	41 (8.4%)				
*Curative treatment*						
No	1397 (67.9%)	116 (8.3%)	**Ref**			
Yes	660 (32.1%)	69 (10.5%)	1.26 (0.93–1.70)	0.130		
Hospital size*						
High volume	1026 (49.9%)	77 (7.5%)	Ref			
Low volume	1031 (50.1%)	108 (10.5%)	1.40 (1.04–1.87)	0.025		
Stenting*						
No	782 (38.0%)	68 (8.7%)	Ref			
Yes	1275 (62.0%)	117 (9.2%)	1.06 (0.78–1.42)	0.724		
Stenosis*						
No	407 (19.8%)	31 (7.6%)	Ref			
Below cystic duct	540 (26.2%)	47 (8.7%)	1.14 (0.73–1.80)	0.564		
Above cystic duct	1110 (54.0%)	107 (9.6%)	1.27 (0.85–1.89)	0.248		

*Not included in multivariate analysis

For the palliative group, patients < 60 years reported 14.5% PEP. In comparison patients age 65–75 years and age > 75% had significantly lower PEP rates at 8.1% (*p* = 0.015) and 6.6% (*p* = 0.003), respectively, in univariate analysis. These differences were significant also in multivariate analysis (Table 3b in supplements).

No difference regarding age was seen for curatively treated patients in uni- or multivariate analysis.

Regarding ASA, no difference was seen for the total cohort or for curative patients.

Palliative patients with ASA score 1 had a PEP rate of 14.8%. In comparison ASA class 2 reported 8.1% (*p* = 0.022) and ASA group 3–4 7.1% (*p* = 0.010) in univariate analysis but no significant difference in multivariate analysis (Table 3b in supplements).

As for size of endoscopy unit there was a higher PEP frequency in larger centers compared to smaller ones (10.5% compared to 7.5% *p* = 0.025) in the total cohort.

### Survival

The 30-day post-ERCP mortality for the total cohort was 8.4%, ranging from 3.5% for dCCA to 13.6% for other CCA (*p* = < 0.001). For the total cohort there was a 90-day mortality of 23.9%, however, with significant differences among the diagnoses with GBC and iCCA mortality rate over 30% but 10.8% for dCCA (Table 2).

**Fig. 3:**
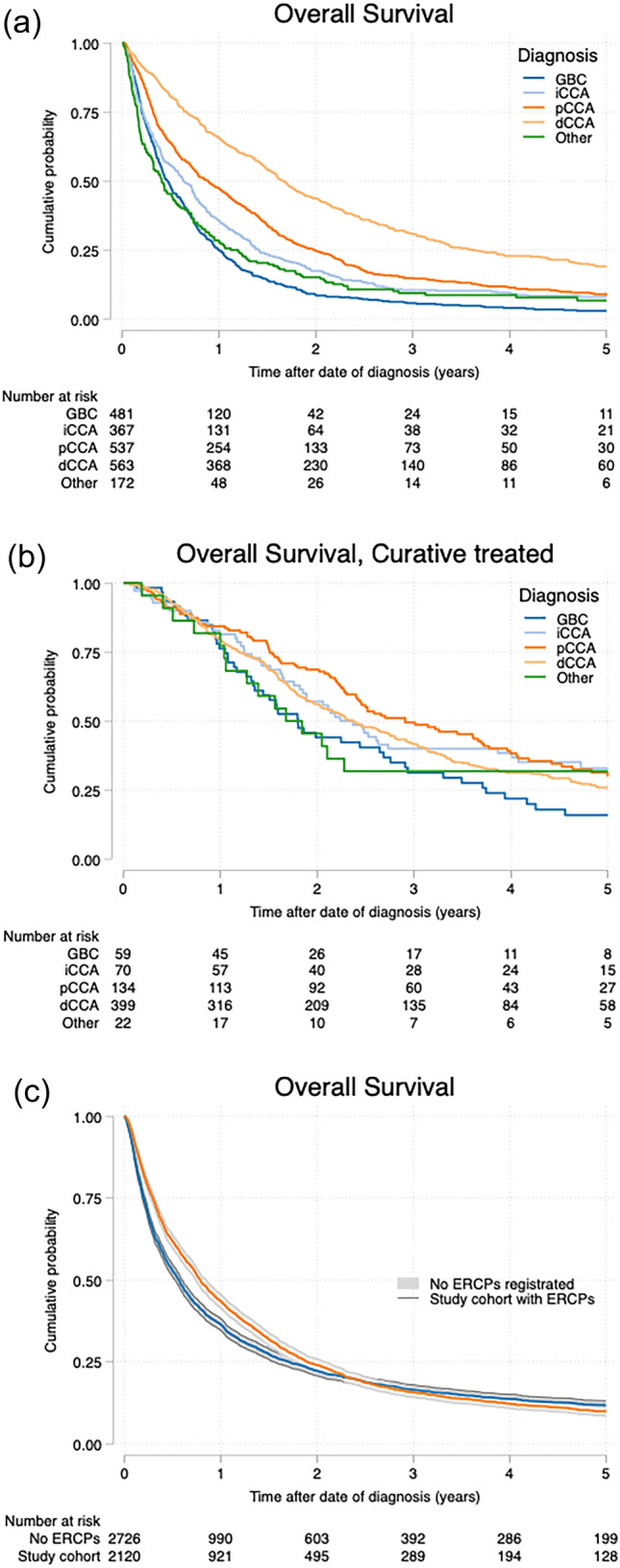
**a** Overall survival ERCP cohort. **b** Overall survival ERCP cohort, curative patients. **c** Overall survival ERCP cohort vs no ERCP

The overall 5-year survival for the total ERCP cohort was 6% ranging from 2–18% depending on diagnosis, see Fig. 3a. For patients treated with curative intention, the range was 14–24%. GBC had the lowest overall survival in both groups (Fig. 3a–b).

There was no statistically significant difference in overall survival between the patients who suffered periprocedural complications after ERCP compared to those that did not.

No difference in survival was seen between the ERCP cohort and the 2726 patients selected from SweLiv and the Pancreas register that did not undergo ERCPs (Fig. 3c).

## Discussion

In this national cohort of patients with only biliary cancers, we studied the use of ERCP during the diagnostic workup (time around date of diagnosis). Many seemed to undergo the procedure for diagnostic purposes, since only about 60% of our cohort did receive a stent. The clinical setting when performing ERCP differ between the biliary cancers. While commonly performed in extrahepatic bile duct cancers (83% of distal bile duct cancer), significantly fewer were performed among GBC and iCCA (around 30%) and for these diagnoses the need for ERCP might reflect perihilar compression or extension. Corresponding to a more advanced situation for many of these patients, 90-day mortality was almost one third for GBC and iCCC.

Endoscopy in malignant hilar obstruction is regarded as technically demanding [17, 18], although biliary cancer is rarely mentioned as cause for increased risk of complications. Often patients with malignancy are regarded as one entity, but in a malignant cohort of patients that undergo ERCP, the biliary cancers often constitute a minority [19]. In order to question if biliary cancers in itself is associated with a high complication rate, we explored this question within a cohort of biliary cancers (2120 patients).

Periprocedural complications were registered in 20% in our cohort of patients with biliary cancers. In comparison, analyzing a cohort with unselected diagnosis, from the same registry in Sweden, a 17% complication rate was found [15], including all postoperative complications like urinary tract infections, etc. (those were excluded in our cohort). In addition, we found statistically significant differences in rates of postprocedural complications between the diagnoses, ranging from 14.8 to 23.3%, where pCCA was the diagnosis with the highest overall complication rate. When analyzing the cohort that did undergo curative treatments, the peak periprocedural complication rate for pCCA was even higher at 30.7% (Table 1a in supplements).

We found a PEP rate of 8.7%. PEP was defined as patients with abdominal pain and s-amylase × 3 at > 24h, requiring hospitalization. All PEP according to this definition were included, not only moderate or severe. Technical risk factors for PEP such as difficult cannulation were not studied in particular but our cohort has a high rate of failed cannulations and interrupted attempts (11%).

There are established risk factors for complications including PEP, where e.g., suspected sphincter Oddi dysfunction and female sex, as well as technical factors such as difficult cannulation are considered factors that increase the risk of PEP. The risk of PEP, based on modern meta-analyses including 18 randomized studies and a large RCT by Lou in 2018 as well as in a recent study, seems to be around 3–6%. For high-risk patients the risk is doubled [20–22]. Studies based on retrospective data seem to report a slightly lower rate of complications than RCTs [2]. In our cohort, younger patients (< 60 years old) suffered more PEP than older patients, and the same tendency was seen in the curative and palliative subgroups. pCCA was the diagnosis with the numerically highest PEP rate in all three groups, with a risk as high as 15% for curatively treated patients.

Overall, our results are in the range of mid- to high-risk patients and biliary cancer patients should be considered as such.

The risk of cholangitis (biliary sepsis) at 6% in this cohort seems unrelated to factors like stricture level, age, sex and locally advanced tumors [23]. These factors seem to be diminished since biliary cancer in itself do carry a higher risk. We found a numerically higher risk of cholangitis for pCCA overall and specifically so in the curative group at about 10%. This may, at least in part, be explained by the complexity of the strictures. A cholangitis rate of 0.5–3% has been reported in mixed cohorts [2] and as high as 21.5% after stenting for malignant biliary obstruction in ERCP naïve patients [24]. Given that malignant strictures, and hilar obstruction, are considered to be risk factors for cholangitis [2, 4] our rate of 6% seems acceptable.

In comparison, a higher complication rate for hilar strictures was also shown by Lubbe et al., in another GallRiks based study, where successful placements of metal stents in patients with malignant hilar obstruction was compared to malignant distal strictures. A post-procedural complication rate (including PEP, cholangitis, perforation, bleeding and other) of about 17% was reported in hilar strictures and 12% in distal [25]. A recent Norwegian study of stenting malignant biliary obstruction presented an endoscopy-related complication rate of 15 and 9% PEP [19].

Our results are in the same level as both above mentioned studies. It should be noted that we did not exclude interrupted procedures, but included all ERCP, not taking stent deployment or technical success into account.

High ASA correlated to significantly less endoscopy-related complications in our cohort, including both PEP and cholangitis. ASA III or higher is regarded an independent risk factor for severe complications (analyzing pancreatitis, bleeding and overall including pulmonary and cardiac complications) and ASA III or less have been shown to suffer less complications [26, 27]. Sedation-related complications are more common in higher ASA and often included in earlier studies, though not included in this study which might affect our results. However, in our cohort it’s important to notice that low ASA do not protect from complications, rather shows an increased risk. For palliative patients, where the indication of ERCP may be to relieve symptoms rather than prolong life, this should be taken into account.

In short, younger and curatively treated patients, suffered a higher complication rate. Whether complications associated with ERCP led to postponed or cancelled surgery is unclear. Though important, in this study we did not have the means to investigate this further. In our cohort about 30% underwent resection, varying between diagnoses, and this high rate may in part be explained by the high percentage of resected distal CCA. With this in mind, we estimate that the number of patients planned for, but ending up non-resected should be fairly low.

### Difference with regard to stage and diagnosis

Since technical difficulty and need of difficulties in stenting could add complexity to the procedure, we anticipated that an advanced tumor stage would increase the risk of complications. However, our results showed no difference in complication risk due to stage.

For patients receiving curatively aiming treatment there was a correlation with a high-risk of complications, and speculatively the technical need for extended examination in and above the hilar region, might impact complication risk to a higher extent than stage.

Differences between diagnoses were non-significant, although pCCA suffered the highest rate of overall periprocedural complications (above 30% and the PEP rate was 15%). For patients with curative intent, also the cholangitis rate was higher for pCCA. Although not statistically significant, these findings show a tendency towards more complications in pCCA patients with curative intent, as well as for younger patients.

In short, younger and curatively treated patients, suffered a higher complication rate. Whether complications associated with ERCP led to postponed or cancelled surgery is unclear. Though important, in this study we did not have the means to investigate this further. In our cohort about 30% underwent resection, varying between diagnoses, and this high rate may in part be explained by the high percentage of resected distal CCA. With this in mind, we estimate that the number of patients planned for, but ending up non-resected should be fairly low.

### Volume

We found a higher complication rate in high volume centers when compared to low volume. The higher complication rate in larger hospitals may be due to case selection or more complex procedures being performed.

Our results indicate that center size in itself, is not a guarantee for decrease in complication rate nor a support for the ongoing discussion of centralization of biliary interventions for malignancy [28, 29]. Experience and the individual skill of the endoscopist seem to impact risk of complications [28–31]. and the European Society of Clinical Gastrointestinal Endoscopy (ESGE) Clinical Guidelines recommends performing drainage of malignant hilar strictures in high volume centers [32]. However, in this study, we did not examine individual skill but only divided centers in higher or lower volume. Supporting our findings, an earlier study from Sweden reported no correlation between center volume and complication risk [33]. Since palliative patients did have a lower complication rate and the need for mapping of tumor extension is less in palliative patients, the possibility to perform ERCP at smaller endoscopy units may still be clinically indicated, in spite of the recommendation by ESGE, and experience by the endoscopists might be more important than center size, although we did not explore this is our study.

### Survival

The 30d and 90d post-ERCP mortality rate of 8.4 and 23.7%, respectively, is in the range of mortality in other studies [34, 35] indicating that ERCP is common even in late palliative setting. Overall survival was not affected by ERCP, when compared to those that did not undergo ERCP, and we do think that although complications for the individual might be serious, the impact on survival for the cohort is minor. Regarding overall survival for curatively treated patients, is important to note that the ERCP cohort for especially GBC and iCCC is a prognostically inferior group of patients (Fig. 3b). Jaundice in patients with gallbladder cancer has long been associated with inferior prognosis, and only 10% of those GBC patients that underwent ERCP did undergo curatively aiming resection. In line, 5-year survival in this group is still inferior.

### Strengths and limitations

The strength of this study is that we did include the total national cohort of biliary cancers.

A limitation is the setting, where register data are inferior to data that is required in a randomized study. Since register data is the only way to get information regarding national cohorts, we do think that our findings should be regarded as relevant.

## Conclusion

About 40% of all biliary cancers underwent ERCP to achieve diagnosis and biliary decompression. The risk of complications in general and especially for pancreatitis seems to be equivalent to a high-risk cohort, which makes evaluation of indication, as well as awareness of complication risk imperative.

Biliary cancer diagnoses differ, and pCCA show a higher risk than other types of biliary cancer. Center volume did not impact risk of complications, and our findings indicate that centralization to centers with higher volume without assessing individual quality of the endoscopist will not be a successful strategy.

Patients with ASA class 3–4 suffered significantly less endoscopy-related complications than patients with lower ASA class, and patients > 75 years old had a lower risk of PEP. No correlation was found regarding sex, or level of stricture. Neither was a locally advanced stage in itself a risk factor.

Overall survival was not affected by ERCP or its complications, but strategies to decrease risks and improve outcome are needed.

## Supplementary Information

Below is the link to the electronic supplementary material.

Table 1a. Intra- and post-procedural complications (30 d), grouped by diagnosis. Curative treated.
Supplementary file1 (DOCX 16 KB)

Table 1b. Intra- and post-procedural complications (30d) by diagnosis. Non curative treated
Supplementary file2 (DOCX 16 KB)

Table 2a. Postprocedural complications, curatively treated
Supplementary file3 (DOCX 15 KB)

Table 2b. Postprocedural complications. Non-curative treated.
Supplementary file4 (DOCX 15 KB)

Table 3. Postprocedural pancreatitis (PEP). Non-curative treated.
Supplementary file5 (DOCX 15 KB)
