# Supplementary material for: Risks and use of ERCP during the diagnostic workup in a national cohort of biliary cancer
Source: Surg Endosc. 2024 Dec 13;39(2):991–1001. doi: 10.1007/s00464-024-11449-8 (PMC11794412; doi:10.1007/s00464-024-11449-8)
Supplement: Supplementary file 1 — Table 1a. Intra- and post-procedural complications (30 d), grouped by diagnosis. Curative treated. Supplementary file1 (DOCX 16 KB) [file 464_2024_11449_MOESM1_ESM.docx]

|  | **GBC** | **iCCA** | **pCCA** | **dCCA** | **Other** | **Total** | **p-value** |
| --- | --- | --- | --- | --- | --- | --- | --- |
|  | **N=56** | **N=69** | **N=127** | **N=386** | **N=22** | **N=660** |  |
| Periprocedural complications |  |  |  |  |  |  | 0.011 |
| No | 42 (75.0%) | 52 (75.4%) | 88 (69.3%) | 319 (82.6%) | 20 (90.9%) | 521 (78.9%) |  |
| Yes | 14 (25.0%) | 17 (24.6%) | 39 (30.7%) | 67 (17.4%) | 2 ( 9.1%) | 139 (21.1%) |  |
| Intraprocedural total |  |  |  |  |  |  | 0.076 |
| No | 53 (94.6%) | 64 (92.8%) | 124 (97.6%) | 379 (98.2%) | 22 (100.0%) | 642 (97.3%) |  |
| Yes | 3 ( 5.4%) | 5 ( 7.2%) | 3 ( 2.4%) | 7 ( 1.8%) | 0 ( 0.0%) | 18 ( 2.7%) |  |
| Intraprocedural bleeding |  |  |  |  |  |  | 0.66 |
| No | 56 (100.0%) | 69 (100.0%) | 126 (99.2%) | 385 (99.7%) | 22 (100.0%) | 658 (99.7%) |  |
| Yes | 0 ( 0.0%) | 0 ( 0.0%) | 1 ( 0.8%) | 1 ( 0.3%) | 0 ( 0.0%) | 2 ( 0.3%) |  |
| Intraprocedural leakage/bile leakage |  |  |  |  |  |  | 0.061 |
| No | 53 (94.6%) | 64 (92.8%) | 125 (98.4%) | 379 (98.2%) | 22 (100.0%) | 643 (97.4%) |  |
| Yes | 3 ( 5.4%) | 5 ( 7.2%) | 2 ( 1.6%) | 7 ( 1.8%) | 0 ( 0.0%) | 17 ( 2.6%) |  |
| Postprocedural complications total |  |  |  |  |  |  | 0.022 |
| No | 45 (80.4%) | 57 (82.6%) | 90 (70.9%) | 324 (83.9%) | 20 (90.9%) | 536 (81.2%) |  |
| Yes | 11 (19.6%) | 12 (17.4%) | 37 (29.1%) | 62 (16.1%) | 2 ( 9.1%) | 124 (18.8%) |  |
| Postprocedural bleeding |  |  |  |  |  |  | 0.079 |
| No | 55 (98.2%) | 67 (97.1%) | 123 (96.9%) | 384 (99.5%) | 22 (100.0%) | 651 (98.6%) |  |
| Yes | 1 ( 1.8%) | 2 ( 2.9%) | 4 ( 3.1%) | 2 ( 0.5%) | 0 ( 0.0%) | 9 ( 1.4%) |  |
| Postprocedural leakage |  |  |  |  |  |  | 0.49 |
| No | 55 (98.2%) | 67 (97.1%) | 123 (96.9%) | 381 (98.7%) | 22 (100.0%) | 648 (98.2%) |  |
| Yes | 1 ( 1.8%) | 2 ( 2.9%) | 4 ( 3.1%) | 5 ( 1.3%) | 0 ( 0.0%) | 12 ( 1.8%) |  |
| Postprocedural pancreatitis (PEP) |  |  |  |  |  |  | 0.52 |
| No | 51 (91.1%) | 63 (91.3%) | 108 (85.0%) | 349 (90.4%) | 20 (90.9%) | 591 (89.5%) |  |
| Yes | 5 ( 8.9%) | 6 ( 8.7%) | 19 (15.0%) | 37 ( 9.6%) | 2 ( 9.1%) | 69 (10.5%) |  |
| Postprocedural cholangitis |  |  |  |  |  |  | 0.11 |
| No | 53 (94.6%) | 67 (97.1%) | 114 (89.8%) | 369 (95.6%) | 22 (100.0%) | 625 (94.7%) |  |
| Yes | 3 ( 5.4%) | 2 ( 2.9%) | 13 (10.2%) | 17 ( 4.4%) | 0 ( 0.0%) | 35 ( 5.3%) |  |
| Postprocedural other |  |  |  |  |  |  | 0.94 |
| No | 54 (96.4%) | 68 (98.6%) | 124 (97.6%) | 374 (96.9%) | 22 (100.0%) | 642 (97.3%) |  |
| Yes | 2 ( 3.6%) | 1 ( 1.4%) | 3 ( 2.4%) | 12 ( 3.1%) | 0 ( 0.0%) | 18 ( 2.7%) |  |
| 30d mortality |  |  |  |  |  |  | 0.84 |
| No | 56 (100.0%) | 69 (100.0%) | 127 (100.0%) | 384 (99.5%) | 22 (100.0%) | 658 (99.7%) |  |
| Yes | 0 ( 0.0%) | 0 ( 0.0%) | 0 ( 0.0%) | 2 ( 0.5%) | 0 ( 0.0%) | 2 ( 0.3%) |  |
| 90d mortality |  |  |  |  |  |  | 0.26 |
| No | 55 (98.2%) | 64 (92.8%) | 122 (96.1%) | 377 (97.7%) | 21 (95.5%) | 639 (96.8%) |  |
| Yes | 1 ( 1.8%) | 5 ( 7.2%) | 5 ( 3.9%) | 9 ( 2.3%) | 1 ( 4.5%) | 21 ( 3.2%) |  |

*Fisher´s exact test.

24 patients with missing values on postprocedural complication are excluded.
