# Supplementary material for: Risks and use of ERCP during the diagnostic workup in a national cohort of biliary cancer
Source: Surg Endosc. 2024 Dec 13;39(2):991–1001. doi: 10.1007/s00464-024-11449-8 (PMC11794412; doi:10.1007/s00464-024-11449-8)
Supplement: Supplementary file 2 — Table 1b. Intra- and post-procedural complications (30d) by diagnosis. Non curative treated Supplementary file2 (DOCX 16 KB) [file 464_2024_11449_MOESM2_ESM.docx]

|  | **GBC** | **iCCA** | **pCCA** | **dCCA** | **Other** | **Total** | **p-value** |
| --- | --- | --- | --- | --- | --- | --- | --- |
|  | **N=410** | **N=290** | **N=389** | **N=161** | **N=147** | **N=1,397** |  |
| Periprocedural complications |  |  |  |  |  |  | 0.76 |
| No | 326 (79.5%) | 233 (80.3%) | 302 (77.6%) | 132 (82.0%) | 120 (81.6%) | 1,113 (79.7%) |  |
| Yes | 84 (20.5%) | 57 (19.7%) | 87 (22.4%) | 29 (18.0%) | 27 (18.4%) | 284 (20.3%) |  |
| Intraprocedural total |  |  |  |  |  |  | 0.12 |
| No | 402 (98.0%) | 279 (96.2%) | 385 (99.0%) | 158 (98.1%) | 142 (96.6%) | 1,366 (97.8%) |  |
| Yes | 8 ( 2.0%) | 11 ( 3.8%) | 4 ( 1.0%) | 3 ( 1.9%) | 5 ( 3.4%) | 31 ( 2.2%) |  |
| Intraprocedural bleeding |  |  |  |  |  |  | 0.16 |
| No | 408 (99.5%) | 287 (99.0%) | 388 (99.7%) | 159 (98.8%) | 144 (98.0%) | 1,386 (99.2%) |  |
| Yes | 2 ( 0.5%) | 3 ( 1.0%) | 1 ( 0.3%) | 2 ( 1.2%) | 3 ( 2.0%) | 11 ( 0.8%) |  |
| Intraprocedural leakage/bile leakage |  |  |  |  |  |  | 0.28 |
| No | 404 (98.5%) | 282 (97.2%) | 386 (99.2%) | 160 (99.4%) | 145 (98.6%) | 1,377 (98.6%) |  |
| Yes | 6 ( 1.5%) | 8 ( 2.8%) | 3 ( 0.8%) | 1 ( 0.6%) | 2 ( 1.4%) | 20 ( 1.4%) |  |
| Postprocedural complications total |  |  |  |  |  |  | 0.50 |
| No | 331 (80.7%) | 238 (82.1%) | 306 (78.7%) | 135 (83.9%) | 124 (84.4%) | 1,134 (81.2%) |  |
| Yes | 79 (19.3%) | 52 (17.9%) | 83 (21.3%) | 26 (16.1%) | 23 (15.6%) | 263 (18.8%) |  |
| Postprocedural bleeding |  |  |  |  |  |  | 0.24 |
| No | 397 (96.8%) | 286 (98.6%) | 385 (99.0%) | 159 (98.8%) | 145 (98.6%) | 1,372 (98.2%) |  |
| Yes | 13 ( 3.2%) | 4 ( 1.4%) | 4 ( 1.0%) | 2 ( 1.2%) | 2 ( 1.4%) | 25 ( 1.8%) |  |
| Postprocedural leakage |  |  |  |  |  |  | 0.84 |
| No | 401 (97.8%) | 282 (97.2%) | 381 (97.9%) | 156 (96.9%) | 145 (98.6%) | 1,365 (97.7%) |  |
| Yes | 9 ( 2.2%) | 8 ( 2.8%) | 8 ( 2.1%) | 5 ( 3.1%) | 2 ( 1.4%) | 32 ( 2.3%) |  |
| Postprocedural pancreatitis (PEP) |  |  |  |  |  |  | 0.80 |
| No | 373 (91.0%) | 270 (93.1%) | 353 (90.7%) | 149 (92.5%) | 136 (92.5%) | 1,281 (91.7%) |  |
| Yes | 37 ( 9.0%) | 20 ( 6.9%) | 36 ( 9.3%) | 12 ( 7.5%) | 11 ( 7.5%) | 116 ( 8.3%) |  |
| Postprocedural cholangitis |  |  |  |  |  |  | 0.69 |
| No | 386 (94.1%) | 272 (93.8%) | 358 (92.0%) | 151 (93.8%) | 140 (95.2%) | 1,307 (93.6%) |  |
| Yes | 24 ( 5.9%) | 18 ( 6.2%) | 31 ( 8.0%) | 10 ( 6.2%) | 7 ( 4.8%) | 90 ( 6.4%) |  |
| Postprocedural other |  |  |  |  |  |  | 0.48 |
| No | 394 (96.1%) | 283 (97.6%) | 376 (96.7%) | 159 (98.8%) | 144 (98.0%) | 1,356 (97.1%) |  |
| Yes | 16 ( 3.9%) | 7 ( 2.4%) | 13 ( 3.3%) | 2 ( 1.2%) | 3 ( 2.0%) | 41 ( 2.9%) |  |
| 30d mortality |  |  |  |  |  |  | 0.036 |
| No | 351 (85.6%) | 250 (86.2%) | 357 (91.8%) | 144 (89.4%) | 124 (84.4%) | 1,226 (87.8%) |  |
| Yes | 59 (14.4%) | 40 (13.8%) | 32 ( 8.2%) | 17 (10.6%) | 23 (15.6%) | 171 (12.2%) |  |
| 90d mortality |  |  |  |  |  |  | <0.001 |
| No | 257 (62.7%) | 184 (63.4%) | 296 (76.1%) | 109 (67.7%) | 80 (54.4%) | 926 (66.3%) |  |
| Yes | 153 (37.3%) | 106 (36.6%) | 93 (23.9%) | 52 (32.3%) | 67 (45.6%) | 471 (33.7%) |  |

*Fisher´s exact test.

39 patients with missing values on postprocedural complication are excluded.
