# Supplementary material for: Risks and use of ERCP during the diagnostic workup in a national cohort of biliary cancer
Source: Surg Endosc. 2024 Dec 13;39(2):991–1001. doi: 10.1007/s00464-024-11449-8 (PMC11794412; doi:10.1007/s00464-024-11449-8)
Supplement: Supplementary file 3 — Table 2a. Postprocedural complications, curatively treated Supplementary file3 (DOCX 15 KB) [file 464_2024_11449_MOESM3_ESM.docx]

|  | **Curative ERCP cohort**  **N=660**  **n (%)** | **Complications N=139**  **n (%)** | **Univariable**  **Poisson regression**  **IRR (CI 95%)** | ***P*** | **Multivariable**  **Poisson regression**  **IRR (CI 95%)** | ***P*** |
| --- | --- | --- | --- | --- | --- | --- |
| **Age group** |  |  |  |  |  |  |
| <60 | 138 (20.9%) | 32 (23.2%) | Ref. |  |  |  |
| 60-75 | 393 (59.5%) | 78 (19.8%) | 0.86 (0.57-1.29) | 0.459 |  |  |
| >75 | 129 (19.5%) | 29 (22.5%) | 0.97 (0.59-1.60) | 0.904 |  |  |
| **Sex** |  |  |  |  |  |  |
| Male | 378 (57.3%) | 84 (22.2%) | Ref. |  |  |  |
| Female | 282 (42.7%) | 55 (19.5%) | 0.88 (0.62-1.23) | 0.452 |  |  |
| **ASA grp** |  |  |  |  |  |  |
| 1 | 120 (18.2%) | 21 (17.5%) | Ref. |  |  |  |
| 2 | 408 (61.8%) | 95 (23.3%) | 1.33 (0.83-2.13) | 0.236 |  |  |
| 3-4 | 132 (20.0%) | 23 (17.4%) | 1.00 (0.55-1.80) | 0.989 |  |  |
| **Diagnosis** |  |  |  |  |  |  |
| GBC | 56 ( 8.5%) | 14 (25.0%) | Ref. |  |  |  |
| ICCA | 69 (10.5%) | 17 (24.6%) | 0.99 (0.49-2.00) | 0.968 |  |  |
| pCCA | 127 (19.2%) | 39 (30.7%) | 1.23 (0.67-2.26) | 0.509 |  |  |
| dCCA | 386 (58.5%) | 67 (17.4%) | 0.69 (0.39-1.24) | 0.214 |  |  |
| Other | 22 ( 3.3%) | 2 ( 9.1%) | 0.36 (0.08-1.60) | 0.181 |  |  |
| **Locally advanced*** |  |  |  |  |  |  |
| No | 192 (29.1%) | 48 (25.0%) | Ref. |  |  |  |
| Yes | 145 (22.0%) | 32 (22.1%) | 0.88 (0.56-1.38) | 0.585 |  |  |
| Missing | 323 (48.9%) | 59 (18.3%) |  |  |  |  |
| **Hospital size*** |  |  |  |  |  |  |
| High volume | 262 (39.7%) | 55 (21.0%) | Ref. |  |  |  |
| Low volume | 398 (60.3%) | 84 (21.1%) | 1.12 (0.81-1.57) | 0.489 |  |  |
| **Stenting*** |  |  |  |  |  |  |
| No | 146 (22.1%) | 31 (21.2%) | Ref. |  |  |  |
| Yes | 292 (44.2%) | 50 (17.1%) | 1.01 (0.72-1.41) | 0.975 |  |  |
| **Stenosis*** | 222 (33.6%) | 58 (26.1%) |  |  |  |  |
| No | 138 (20.9%) | 32 (23.2%) | Ref. |  |  |  |
| Below cystic duct | 393 (59.5%) | 78 (19.8%) | 0.81 (0.52-1.26) | 0.347 |  |  |
| Above cystic duct | 129 (19.5%) | 29 (22.5%) | 1.23 (0.80-1.90) | 0.351 |  |  |

### * not included in multivariate analysis
