# Supplementary material for: Risks and use of ERCP during the diagnostic workup in a national cohort of biliary cancer
Source: Surg Endosc. 2024 Dec 13;39(2):991–1001. doi: 10.1007/s00464-024-11449-8 (PMC11794412; doi:10.1007/s00464-024-11449-8)
Supplement: Supplementary file 4 — Table 2b. Postprocedural complications. Non-curative treated. Supplementary file4 (DOCX 15 KB) [file 464_2024_11449_MOESM4_ESM.docx]

|  | **N=1,397**  **n (%)** | | **Number of**  **POCs N=284**  **n (%)** | **Univariable**  **Poisson regression**  **IRR (CI 95%)** | ***P*** | **Multivariable**  **Poisson regression**  **IRR (CI 95%)** | ***P*** |
| --- | --- | --- | --- | --- | --- | --- | --- |
| **Age group** | |  |  |  |  |  |  |
| <60 | | 172 (12.3%) | 40 (23.3%) | Ref. |  |  |  |
| 60-75 | | 681 (48.7%) | 140 (20.6%) | 0.88 (0.62-1.26) | 0.492 |  |  |
| >75 | | 544 (38.9%) | 104 (19.1%) | 0.82 (0.57-1.18) | 0.292 |  |  |
| **Sex** | |  |  |  |  |  |  |
| Male | | 611 (43.7%) | 124 (20.3%) | Ref. |  |  |  |
| Female | | 786 (56.3%) | 160 (20.4%) | 1.00 (0.79-1.27) | 0.980 |  |  |
| **ASA grp** | |  |  |  |  |  |  |
| 1 | | 128 ( 9.2%) | 42 (32.8%) | Ref. |  |  |  |
| 2 | | 694 (49.7%) | 142 (20.5%) | 0.62 (0.44-0.88) | 0.007 |  |  |
| 3-4 | | 575 (41.2%) | 100 (17.4%) | 0.53 (0.37-0.76) | 0.001 |  |  |
| **Diagnosis** | |  |  |  |  |  |  |
| GBC | | 410 (29.3%) | 84 (20.5%) | Ref. |  |  |  |
| ICCA | | 290 (20.8%) | 57 (19.7%) | 0.96 (0.69-1.34) | 0.809 |  |  |
| pCCA | | 389 (27.8%) | 87 (22.4%) | 1.09 (0.81-1.47) | 0.567 |  |  |
| dCCA | | 161 (11.5%) | 29 (18.0%) | 0.88 (0.58-1.34) | 0.550 |  |  |
| Other | | 147 (10.5%) | 27 (18.4%) | 0.90 (0.58-1.38) | 0.621 |  |  |
| **Locally advanced*** | |  |  |  |  |  |  |
| No | | 431 (30.9%) | 90 (20.9%) | Ref. |  |  |  |
| Yes | | 801 (57.3%) | 154 (19.2%) | 0.92 (0.71-1.19) | 0.534 |  |  |
| Missing | | 165 (11.8%) | 40 (24.2%) |  |  |  |  |
| **Hospital size*** | |  |  |  |  |  |  |
| High volume | | 698 (50.0%) | 127 (18.2%) | Ref. |  |  |  |
| Low volume | | 699 (50.0%) | 157 (22.5%) | 1.23 (0.98-1.56) | 0.078 |  |  |
| **Stenting*** | |  |  |  |  |  |  |
| No | | 520 (37.2%) | 111 (21.3%) | Ref. |  |  |  |
| Yes | | 877 (62.8%) | 173 (19.7%) | 0.92 (0.73-1.17) | 0.516 |  |  |
| **Stenosis*** | |  |  |  |  |  |  |
| No | | 261 (18.7%) | 57 (21.8%) | Ref. |  |  |  |
| Below cystic duct | | 248 (17.8%) | 47 (19.0%) | 0.87 (0.59-1.28) | 0.472 |  |  |
| Above cystic duct | | 888 (63.6%) | 180 (20.3%) | 0.93 (0.69-1.25) | 0.624 |  |  |

### * not included in multivariate analysis
