# Supplementary material for: Risks and use of ERCP during the diagnostic workup in a national cohort of biliary cancer
Source: Surg Endosc. 2024 Dec 13;39(2):991–1001. doi: 10.1007/s00464-024-11449-8 (PMC11794412; doi:10.1007/s00464-024-11449-8)
Supplement: Supplementary file 5 — Table 3. Postprocedural pancreatitis (PEP). Non-curative treated. Supplementary file5 (DOCX 15 KB) [file 464_2024_11449_MOESM5_ESM.docx]

|  | **N=1,397**  **n (%)** | **Number of**  **POCs N=116**  **n (%)** | **Univariable**  **Poisson regression**  **IRR (CI 95%)** | ***P*** | **Multivariable**  **Poisson regression**  **IRR (CI 95%)** | ***P*** |
| --- | --- | --- | --- | --- | --- | --- |
| **Age group** |  |  |  |  |  |  |
| <60 | 172 (12.3%) | 25 (14.5%) | Ref. |  | Ref. |  |
| 60-75 | 681 (48.7%) | 55 ( 8.1%) | 0.56 (0.35-0.89) | 0.015 | 0.60 (0.37-0.97) | 0.038 |
| >75 | 544 (38.9%) | 36 ( 6.6%) | 0.46 (0.27-0.76) | 0.003 | 0.52 (0.30-0.88) | 0.014 |
| **Sex** |  |  |  |  |  |  |
| Male | 611 (43.7%) | 49 ( 8.0%) | Ref. |  |  |  |
| Female | 786 (56.3%) | 67 ( 8.5%) | 1.06 (0.74-1.54) | 0.745 |  |  |
| **ASA grp** |  |  |  |  |  |  |
| 1 | 128 ( 9.2%) | 19 (14.8%) | Ref. |  | Ref. |  |
| 2 | 694 (49.7%) | 56 ( 8.1%) | 0.54 (0.32-0.91) | 0.022 | 0.62 (0.37-1.06) | 0.081 |
| 3-4 | 575 (41.2%) | 41 ( 7.1%) | 0.48 (0.28-0.83) | 0.008 | 0.57 (0.32-1.00) | 0.052 |
| **Diagnosis** |  |  |  |  |  |  |
| GBC | 410 (29.3%) | 37 ( 9.0%) | Ref. |  |  |  |
| iCCA | 290 (20.8%) | 20 ( 6.9%) | 0.76 (0.44-1.32) | 0.333 |  |  |
| pCCA | 389 (27.8%) | 36 ( 9.3%) | 1.03 (0.65-1.62) | 0.914 |  |  |
| dCCA | 161 (11.5%) | 12 ( 7.5%) | 0.83 (0.43-1.58) | 0.565 |  |  |
| Other | 147 (10.5%) | 11 ( 7.5%) | 0.83 (0.42-1.63) | 0.585 |  |  |
| **Locally advanced*** |  |  |  |  |  |  |
| No | 431 (30.9%) | 35 ( 8.1%) | Ref. |  |  |  |
| Yes | 801 (57.3%) | 70 ( 8.7%) | 1.08 (0.72-1.61) | 0.723 |  |  |
| Missing | 165 (11.8%) | 11 ( 6.7%) |  |  |  |  |
| **Hospital size*** |  |  |  |  |  |  |
| High volume | 698 (50.0%) | 49 ( 7.0%) | Ref. |  |  |  |
| Low volume | 699 (50.0%) | 67 ( 9.6%) | 1.37 (0.94-1.97) | 0.098 |  |  |
| **Stenting*** |  |  |  |  |  |  |
| No | 520 (37.2%) | 44 ( 8.5%) | Ref. |  |  |  |
| Yes | 877 (62.8%) | 72 ( 8.2%) | 0.97 (0.67-1.41) | 0.875 |  |  |
| **Stenosis*** |  |  |  |  |  |  |
| No | 261 (18.7%) | 18 ( 6.9%) | Ref. |  |  |  |
| Below cystic duct | 248 (17.8%) | 18 ( 7.3%) | 1.05 (0.55-2.02) | 0.878 |  |  |
| Above cystic duct | 888 (63.6%) | 80 ( 9.0%) | 1.31 (0.78-2.18) | 0.306 |  |  |

* not included in multivariate analysis
